# Elevated brain natriuretic peptide levels in chronic fatigue syndrome associate with cardiac dysfunction: a case control study

**DOI:** 10.1136/openhrt-2017-000697

**Published:** 2017-12-27

**Authors:** Cara Tomas, Andreas Finkelmeyer, Tim Hodgson, Laura MacLachlan, Guy A MacGowan, Andrew M Blamire, Julia L Newton

**Affiliations:** 1 Institute of Cellular Medicine, Newcastle University, Newcastle, Newcastle upon Tyne, UK; 2 Newcastle Magnetic Resonance Centre, Newcastle University, Newcastle, Newcastle upon Tyne, UK; 3 Cardiology, Newcastle upon Tyne Hospitals NHS Foundation Trust, Newcastle, Newcastle upon Tyne, UK; 4 CRESTA, Newcastle upon Tyne Hospitals NHS Foundation Trust, Newcastle, Newcastle upon Tyne, UK

**Keywords:** fatigue, bnp, cardiac function

## Abstract

**Objectives:**

To explore levels of the brain natriuretic peptide (BNP) and how these associate with the cardiac abnormalities recently identified in chronic fatigue syndrome (CFS).

**Methods:**

Cardiac magnetic resonance examinations were performed using 3T Philips Intera Achieva scanner (Best, Netherlands) in CFS (Fukuda) participants and sedentary controls matched group wise for age and sex. BNP was also measured by using an enzyme immunoassay in plasma from 42 patients with CFS and 10 controls.

**Results:**

BNP levels were significantly higher in the CFS cohort compared with the matched controls (P=0.013). When we compared cardiac volumes (end-diastolic and end-systolic) between those with high BNP levels (BNP >400 pg/mL) and low BNP (<400 pg/mL), there were significantly lower cardiac volumes in those with the higher BNP levels in both end-systolic and end-diastolic volumes (P=0.05). There were no relationships between fatigue severity, length of disease and BNP levels (P=0.2) suggesting that our findings are unlikely to be related to deconditioning.

**Conclusion:**

This study confirms an association between reduced cardiac volumes and BNP in CFS. Lack of relationship between length of disease suggests that findings are not secondary to deconditioning. Further studies are needed to explore the utility of BNP to act as a stratification paradigm in CFS that directs targeted treatments.

**Trail registration number:**

Registered with NIHR Portfolio CLRN ID 97805.

**Key questions**

**What is already known about this subject?**

- Structural and functional cardiac abnormalities have been reported in chronic fatigue syndrome (CFS).
- Magnetic resonance spectroscopy studies have suggested a subclinical cardiomyopathy in some of those with CFS.

**What does this study add?**

- Brain natriuretic peptide (BNP) levels were significantly higher in CFS compared with matched controls.
- There were significantly lower cardiac volumes in those with higher BNP levels in both end-systolic and end-diastolic volumes.
- There were no relationships between fatigue severity, length of illness and BNP levels confirming that our findings are unlikely to be related to deconditioning.

**How might this impact on clinical practice?**

- This study confirms an association between reduced cardiac volumes and BNP in CFS. Lack of relationship between length of disease suggests that findings are not secondary to deconditioning. Further studies are needed to explore the utility of BNP to act as a stratification paradigm in CFS that directs targeted treatments.

## Introduction

Studies performed using a range of assessment modalities have shown that chronic fatigue syndrome (CFS) is associated with abnormalities of cardiac function.^1–6^ Echocardiographic and impedance studies have confirmed impaired cardiac contractility^1 2^ and reduced left ventricular function.^6^ Structural cardiac magnetic resonance (MR) has shown reduced end diastolic dimensions and cardiac output with MR spectroscopy detecting impaired cardiac bioenergetic function^3 4^ with findings suggestive of a subclinical cardiomyopathy in approximately a third of the CFS cohort (ref). The severity of these cardiac abnormalities also appears to relate to symptom severity but does not appear to be secondary to deconditioning.^1 3 5–7^ This has led to the suggestion that CFS is a small heart syndrome^8 9^ with MR findings consistent, in some patients with CFS, with cardiac failure picture.

Brain natriuretic peptide (BNP) is a 32 amino acid polypeptide secreted by the ventricles of the heart in response to excessive stretching of heart muscle cells. BNP has been shown to be a useful screening and prognostic tool in patients with heart failure and is typically found to be increased in patients with left ventricular dysfunction, with or without symptoms.^10–12^

The physiological actions of BNP include decrease in systemic vascular resistance and central venous pressure as well as an increase in natriuresis. The net effect of these peptides is a decrease in blood pressure due to the decrease in systemic vascular resistance and thus after load. Additionally the actions of BNP result in a decrease in cardiac output due to an overall decrease in central venous pressure and preload as a result of a reduction in blood volume that follows natriuresis and diuresis. The utility of BNP as a diagnostic and prognostic stratification factor in patients with heart failure has been studied extensively.^12^

The purpose of this study was therefore to measure BNP levels in patients with CFS compared with controls and to determine whether BNP levels associated with impaired cardiac function.

## Methods

### Subjects

Participants were recruited as part of an observational study aimed at understanding the pathogenesis of autonomic dysfunction in patients with CFS. The recruitment to this study has previously been reported.^6^ Participants fulfilled the diagnostic criteria for CFS.^13^ In order to fulfil these criteria, individuals were required to have no comorbidity including normal renal blood tests and a normal BMI. Participants were not selected positively or negatively according to any criteria other than the fact that they were attending a clinical service and had a Fukuda diagnosis of CFS,^14^ although they were excluded if they screened positive for a major depressive episode as assessed using the Structured Clinical Interview for the Diagnostic and Statistical Manual for Mental Disorders (version IV; SCID-IV^15^). Fatigue impact was assessed by the Fatigue Impact Scale.^16^

Controls were recruited via notices provided in the hospital and University together with a distribution of posters via the local Patient Support Group where we invited relatives of those with CFS to participate. Controls fulfilled the same inclusion and exclusion criteria as CFS participants, and they were sedentary but otherwise not positively or negatively recruited according to fatigue severity or the presence or absence of particular symptoms. All participants provided written informed consent.

### Measurement of brain natriuretic peptide (BNP)

BNP was measured by a researcher blinded to the group for each individual sample, using the brain natriuretic peptide EIA Kit from Sigma Aldrich (RAB0386). Each component of the kit was reconstituted and diluted as directed by the manufacturer. Anti-BNP antibody was added to each well on the BNP microplate and incubated for 1.5 hours at room temperature with gentle shaking (1–2 cycles/s). The solution was discarded and each well washed thoroughly four times, ensuring complete removal of liquid after each wash. Standards and samples were added to the microplate. Standards with known concentrations of BNP were created from BNP standard included in the kit. Twofold dilutions of each plasma sample were created by the addition of an equal volume of biotinylated BNP peptide to the sample. All standards and samples were run in duplicate. The microplate was incubated for 2.5 hours at room temperature with gently shaking. The solution was discarded and washed again as described previously. HRP-streptavidin solution was added to each well and the microplate incubated for 45 min at room temperature with gently shaking. The solution was discarded and washed as described previously. TMB one-step substrate reagent was added to each well and incubated for 30 min at room temperature, in the dark, with gentle shaking. Stop solution was added to each well and the absorbance read on a Tecan infinite M200 plate reader at 450 nm. A standard curve was created using the standards. The BNP concentration in each sample was determined using the standard curve.

This experiment also included a positive control to verify the components of the kit are working correctly.

We considered a BNP value of >400 pg/mL as being consistent with moderate to severe cardiac disease and this was defined prehoc.

### Cardiac MR

Cardiac examinations were performed using a 3T Philips Intera Achieva scanner (Best, Netherlands). A dedicated 6-channel cardiac coil (Philips, Best, Netherlands) is used with the subjects in a supine position and ECG gating (Philips vectorcardiogram, VCG system). Cardiac MR cine imaging is acquired to assess cardiac morphology and systolic and diastolic function. A stack of balanced steady-state free precession images was obtained in the short axis view during breath holding covering the entire left ventricle (FO=350 mm, TR/TE=3.7/1.9 ms, turbo factor 17, flip angle 40°, slice thickness 8 mm, 0 mm gap, 14 slices, 25 phases, resolution 1.37 mm, temporal duration approx. 40 ms per phase, dependent on heart rate). Image analysis was performed using the cardiac analysis package of the ViewForum workstation (Philips, Best, Netherlands). Manual tracing of the epicardial and endocardial borders was performed on the short axis slices at end-systole and end-diastole by a trained radiographer. The algorithm for contour selection and subsequently calculating left ventricular mass, systolic and diastolic parameters have been detailed elsewhere.^17^

### Statistical analysis

Continuous variables were expressed as mean±SD and comparisons made using unpaired t-tests where groups are matched. Correlation analysis was performed using non-parametric testing. Analysis was performed using Graphpad, Prism. Multivariate analysis was performed using SPSS. A statistically significant result was when P <0.05.

## Results

Cardiac MR and BNP were measured in 42 patients with CFS and 10 sedentary controls-matched group wise for age and sex. Length of history for the patients with CFS was mean 13.8 years (SD 9.8). Cardiac MR measurements for the two groups are shown in table 1.

**Table 1:** Cardiac magnetic resonance parameters in CFS compared with matched control values expressed as mean (SD) unless stated

	Controls	CFS		
		Total	BNP >400	BNP <400
N	10	42	21	21
Age (years)	46 (13)	46 (12)	46 (11)	48 (12)
Females (%)	8 (80%)	32 (76%)	15 (71%)	17 (81%)
Fatigue Impact Scale	N/A	92 (34)	89 (32)	95 (36)
Ejection fraction (%)	62 (5.4)	63 (5.1)	64 (6)	63 (4)
Stroke volume (mL)	60 (10)	57 (13)	54 (12)	60 (13)
ED volume (mL)	96 (14.4)	91 (21.4)	85 (20)	95 (20)
ES phase (ms)	327 (47)	320 (48)	308 (33)	336 (58)
ES volume (mL)	37 (8)	34 (10)	31 (10)	35 (8)
ED wall mass (g)	72 (13)	70 (19)	72 (18)	68 (20)
ED wall+Pap mass (g)	80 (13)	77 (21)	80 (20)	75 (22)

BNP, brain natriuretic peptide; CFS, chronic fatigue syndrome; ED, end diastolic; ES, end systolic.

**Figure 1:**
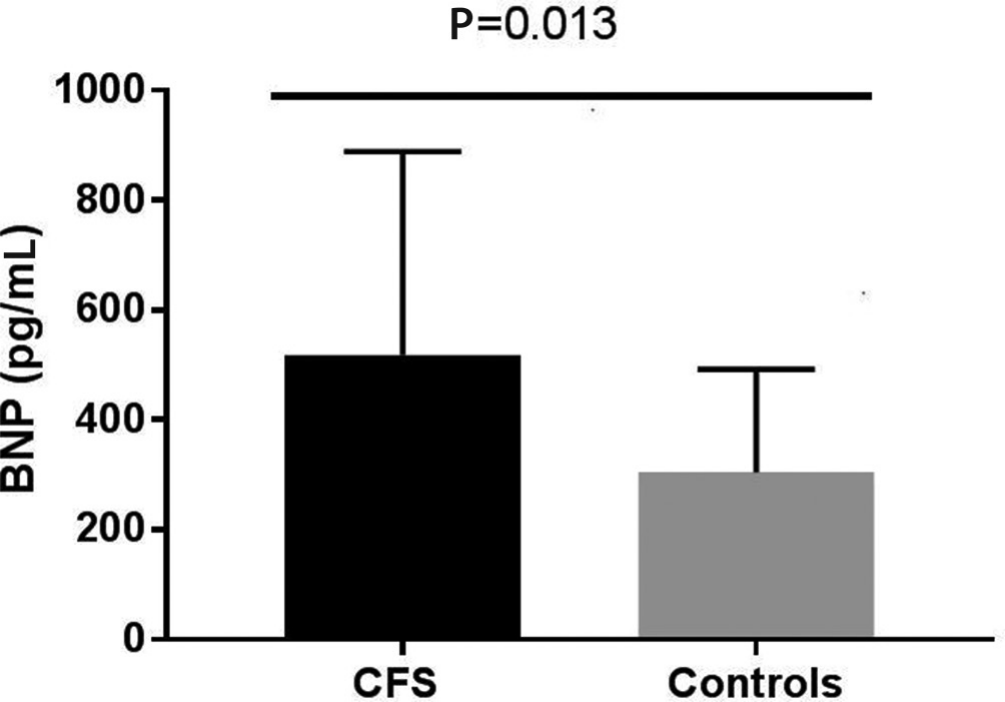
BNP levels were significantly higher in the CFS cohort compared with the matched controls. BNP, brain natriuretic peptide; CFS, chronic fatigue syndrome.

**Figure 2:**
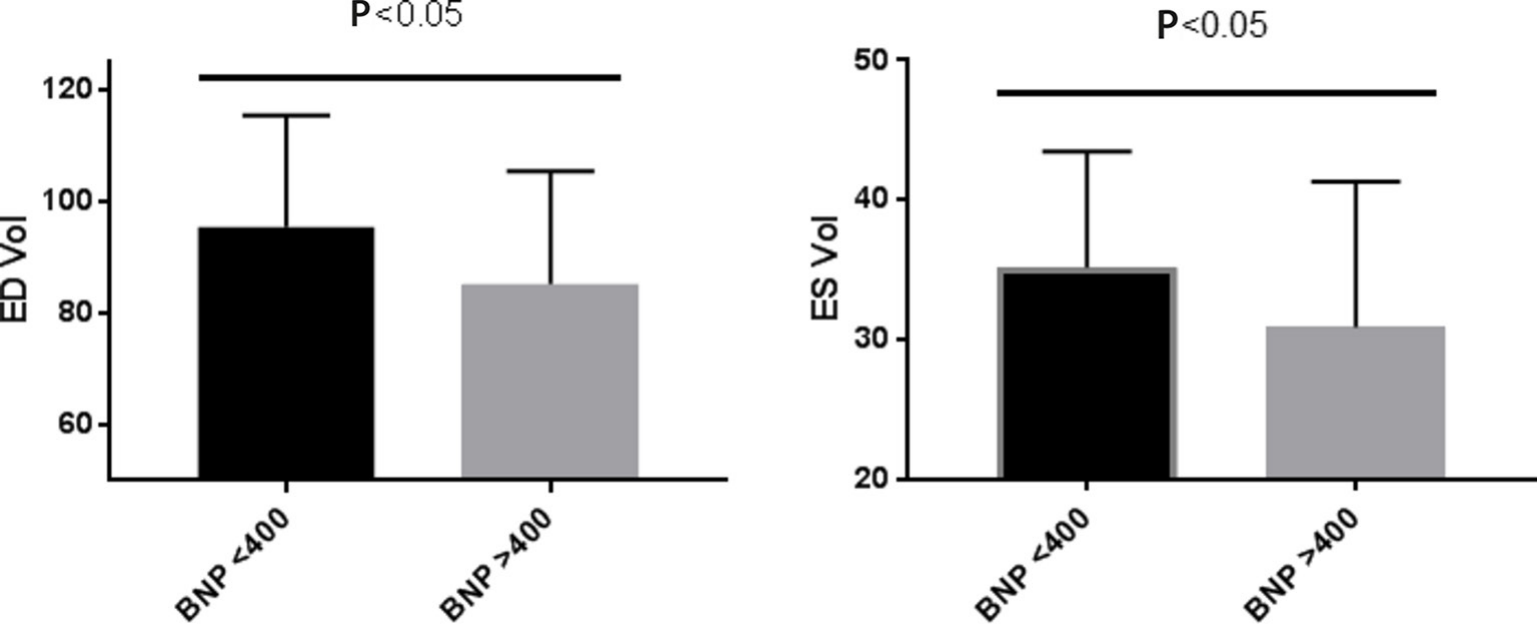
When we compared cardiac volumes (ED and ES) between those with high BNP levels (BNP >400 pg/mL) and low BNP (<400 pg/mL), there were significantly lower cardiac volumes in those with the higher BNP levels in both ES and ED volumes. BNP, brain natriuretic peptide; CFS, chronic fatigue syndrome; ED, end diastolic; ES, end systolic.

BNP levels were significantly higher in the CFS cohort compared with the matched controls (figure 1). When we compared cardiac volumes (end-diastolic and end-systolic) between those with high BNP levels (BNP >400 pg/mL) and low BNP (<400 pg/mL), there were significantly lower cardiac volumes in those with the higher BNP levels in both end-systolic and end-diastolic volumes (figure 2). There were no differences in age, fatigue severity or length of history between the two groups (table 1).

There were no relationships between fatigue severity, length of history and BNP levels (P=0.2).

## Discussion

Studies have confirmed in a range of conditions that BNP can predict prognosis and detect those with cardiac failure. This study has shown that in patients with CFS, a group shown previously to have high levels of subclinical cardiac abnormalities,^1–9^ that BNP is elevated. Studies have also concluded that those with CFS have reduced cardiac volumes, the degree of which associates with plasma volume.^6^ In the present study, higher BNP levels were also shown to be associated with smaller cardiac volumes. The lack of relationship between length of disease and BNP levels suggests that our findings are unlikely to be secondary to deconditioning.

The association found in this study is interesting. It is possible that the smaller cardiac volumes seen in those with CFS are causing the elevated BNP levels. However, this is counterintuitive, and BNP is usually a sign of cardiac ventricular wall strain/stretch and volume overload. In our study, the BNP was higher in the group with the lower cardiac volumes. Another explanation is that the higher BNP levels are causing a diuresis (or natriuresis) and that this is depleting the plasma/blood volumes and leading to the smaller cardiac volumes. Studies from our group and others have shown smaller plasma volumes in CFS^6 7^ and studies with patients with orthostatic hypotension have reported high BNP levels in some patients^18^ and have been suggested as potentially causative.

We believe that measurement of BNP could represent a tool to identify the 1/3 of patients with CFS who were found in previous studies to have impaired cardiac bioenergetic function. Doing this could potentially stratify patients with CFS to more appropriate interventions and also facilitate research to identify the particular characteristics of a cardiac phenotype within the overall cohort with the diagnosis of CFS. We believe that this kind of stratified approach to identifying specific phenotypes and facilitating targeted interventions is an important step in our understanding of the heterogeneous nature of those with CFS.

This study confirms an association between reduced cardiac volumes and BNP in CFS. Lack of relationship between length of disease suggests that findings are not secondary to deconditioning. Further studies are needed to explore the utility of BNP to act as a stratification paradigm in CFS that directs targeted treatments.

## References

[1] Peckerman A, LaManca JJ, Dahl KA, et al Abnormal impedance cardiography predicts symptom severity in chronic fatigue syndrome. Am J Med Sci 2003;326:55–60. 10.1097/00000441-200308000-00001 12920435

[2] LaManca JJ, Peckerman A, Walker J, et al Cardiovascular response during head-up tilt in chronic fatigue syndrome. Clin Physiol 1999;19:111–20. 10.1046/j.1365-2281.1999.00154.x 10200892

[3] Hollingsworth KG, Jones DE, Taylor R, et al Impaired cardiovascular response to standing in chronic fatigue syndrome. Eur J Clin Invest 2010;40:608–15. 10.1111/j.1365-2362.2010.02310.x 20497461

[4] Hollingsworth KG, Hodgson T, Macgowan GA, et al Impaired cardiac function in chronic fatigue syndrome measured using magnetic resonance cardiac tagging. J Intern Med 2012;271:264–70. 10.1111/j.1365-2796.2011.02429.x 21793948 PMC3627316

[5] Hurwitz BE, Coryell VT, Parker M, et al Chronic fatigue syndrome: illness severity, sedentary lifestyle, blood volume and evidence of diminished cardiac function. Clin Sci 2009;118:125–35. 10.1042/CS20090055 19469714

[6] Newton JL, Finkelmeyer A, Petrides G, et al Blamire a reduced cardiac volumes in chronic fatigue syndrome associate with plasma volume but not length of disease: a cohort study. openhrt 2016:e000381. 10.1136/openhrt-2015-000381 PMC4932290 27403329

[7] Farquhar WB, Hunt BE, Taylor JA, et al Blood volume and its relation to peak O(2) consumption and physical activity in patients with chronic fatigue. Am J Physiol Heart Circ Physiol 2002;282:H66–71. 11748048 10.1152/ajpheart.2002.282.1.H66

[8] Miwa K, Fujita M Cardiac function fluctuates during exacerbation and remission in young adults with chronic fatigue syndrome and “small heart”. J Cardiol 2009;54:29–35. 10.1016/j.jjcc.2009.02.008 19632517

[9] Miwa K, Fujita M Small heart syndrome in patients with chronic fatigue syndrome. Clin Cardiol 2008;31:328–33. 10.1002/clc.20227 18636530 PMC6653127

[10] Davidson NC, Struthers AD Brain natriuretic peptide. J Hypertens 1994;12:329–36. 10.1097/00004872-199404000-00001 8064155

[11] Troughton RW, Frampton CM, Yandle TG, et al Treatment of heart failure guided by plasma aminoterminal brain natriuretic peptide (N-BNP) concentrations. Lancet 2000;355:1126–30. 10.1016/S0140-6736(00)02060-2 10791374

[12] Jourdain P, Jondeau G, Funck F, et al Plasma brain natriuretic peptide-guided therapy to improve outcome in heart failure: the STARS-BNP Multicenter Study. J Am Coll Cardiol 2007;49:1733–9. 10.1016/j.jacc.2006.10.081 17448376

[13] Chronic Fatigue Syndrome/Myalgic Encephalomyelitis (encephalopathy); diagnosis and management. www.nice.gov.org

[14] Fukuda K The chronic fatigue syndrome: a comprehensive approach to its definition and study. Ann Intern Med 1994;121:953–9. 10.7326/0003-4819-121-12-199412150-00009 7978722

[15] Pence BW, Miller WC, Gaynes BN Prevalence estimation and validation of new instruments in psychiatric research: an application of latent class analysis and sensitivity analysis. Psychol Assess 2009;21:235–9. 10.1037/a0015686 19485679 PMC2855555

[16] Fisk JD, Ritvo PG, Ross L, et al Measuring the functional impact of fatigue: initial validation of the fatigue impact scale. Clin Infect Dis 1994;18(Suppl 1):S79–83. 10.1093/clinids/18.Supplement_1.S79 8148458

[17] Schar M, Kozerke S, Harvey PR, et al Local linear shimming for cardiac SSFP imaging at 3T. Proc. ISMRM 2002;10:1735.

[18] Krishnan B, Patarroyo-Aponte M, Duprez D, et al Orthostatic hypotension of unknown cause: unanticipated association with elevated circulating N-terminal brain natriuretic peptide (NT-proBNP). Heart Rhythm 2015;12:1287–94. 10.1016/j.hrthm.2015.02.015 25684232

